# Clinical characteristics and drug resistance of *Nocardia* in Henan, China, 2017–2023

**DOI:** 10.1186/s12941-024-00677-4

**Published:** 2024-03-06

**Authors:** Yungang Han, Meijin Cheng, Zheng Li, Huihui Chen, Shuang Xia, Yue Zhao, Yali Wang, Wenyi He, Wei Wang

**Affiliations:** 1 Key Laboratory of Medical Laboratory, Henan Provincial Chest Hospital, Affiliated Chest Hospital of Zhengzhou University, Zhengzhou, China; 2 Henan Provincial Medical Key Disciplines (Laboratory Diagnostics), Henan Provincial Chest Hospital, Zhengzhou, China

**Keywords:** *Nocardia*, Identification, Drug susceptibility testing, Treatment, Underlying conditions, Bronchiectasis

## Abstract

**Background:**

The aim of this study was to investigate the clinical features of *Nocardia* infections, antibiotic resistance profile, choice of antibiotics and treatment outcome, among others. In addition, the study compared the clinical and microbiological characteristics of nocardiosis in bronchiectasis patients and non-bronchiectasis patients.

**Methods:**

Detailed clinical data were collected from the medical records of 71 non-duplicate nocardiosis patients from 2017 to 2023 at a tertiary hospital in Zhengzhou, China. *Nocardia* isolates were identified to the species level using MALDI-TOF MS and 16S rRNA PCR sequencing. Clinical data were collected from medical records, and drug susceptibility was determined using the broth microdilution method.

**Results:**

Of the 71 cases of nocardiosis, 70 (98.6%) were diagnosed as pulmonary infections with common underlying diseases including bronchiectasis, tuberculosis, diabetes mellitus and chronic obstructive pulmonary disease (COPD). Thirteen different strains were found in 71 isolates, the most common of which were *N. farcinica* (26.8%) and *N. cyriacigeorgica* (18.3%). All *Nocardia* strains were 100% susceptible to both TMP-SMX and linezolid, and different *Nocardia* species showed different patterns of drug susceptibility in vitro. Pulmonary nocardiosis is prone to comorbidities such as bronchiectasis, diabetes mellitus, COPD, etc., and *Nocardia* is also frequently accompanied by co-infection of the body with pathogens such as *Mycobacterium* and *Aspergillus spp*. Sixty-one patients underwent a detailed treatment regimen, of whom 32 (52.5%) received single or multi-drug therapy based on TMP-SMX. Bronchiectasis was associated with a higher frequency of *Nocardia* infections, and there were significant differences between the bronchiectasis and non-bronchiectasis groups in terms of age distribution, clinical characteristics, identification of *Nocardia* species, and antibiotic susceptibility (P < 0.05).

**Conclusions:**

Our study contributes to the understanding of the species diversity of *Nocardia* isolates in Henan, China, and the clinical characteristics of patients with pulmonary nocardiosis infections. Clinical and microbiologic differences between patients with and without bronchiectasis. These findings will contribute to the early diagnosis and treatment of patients.

**Supplementary Information:**

The online version contains supplementary material available at 10.1186/s12941-024-00677-4.

## Introduction

*Nocardia*, a Gram-positive filamentous bacterium, is commonly found in soil, water, and air [1, 2]. It can enter the body through inhalation of hyphal fragments, broken skin, or the digestive tract, leading to pneumonia, brain abscesses, and skin and soft tissue infections [1]. Hosts with suppressed immune function, such as those on long-term glucocorticoid therapy, patients undergoing radiotherapy and chemotherapy for malignant tumors, organ transplantation and hematopoietic stem cell transplantation recipients, and individuals infected with human immunodeficiency virus (HIV), often develop primary pulmonary infections [3]. Non-immunosuppressed hosts primarily suffer from structural lung diseases like cystic fibrosis and bronchiectasis [4, 5].The distribution of *Nocardia* species varies geographically [3]. There is limited research on the clinical characteristics of nocardiosis in mainland China, particularly regarding patient treatment, prognosis, species identification of *Nocardia*, and drug susceptibility testing [6, 7]. To address this knowledge gap, we conducted a retrospective study, collecting detailed clinical data on nocardiosis patients in a tertiary hospital in Henan over a 6-year period. Additionally, we performed species identification of all included *Nocardia* isolates using matrix-assisted laser desorption ionization time-of-flight mass spectrometry (MALDI-TOF MS) and genetic sequencing, as well as tested their antibiotic susceptibility spectrum. In recent years, the prevalence of pulmonary nocardiosis has significantly increased among patients with bronchiectasis. Although the exact reasons for this increase are not fully understood, they may be attributed to environmental exposure and microbial surveillance, among other factors [5]. Therefore, our study aimed to compare the clinical and microbiological characteristics between nocardiosis patients with and without bronchiectasis. To our knowledge, no studies comparing the characteristics of these two patient groups have been conducted in mainland China.

## Materials and methods

### Collection of bacterial strains

We conducted a retrospective analysis of 71 unique cases of *Nocardia* infection that occurred at Henan Chest Hospital from January 2017 to April 2023. These cases involved patients from 14 different cities in Henan Province. Patient medical records were collected, including demographic information, underlying diseases, co-infections, imaging data, laboratory data, antimicrobial treatment, and prognosis. The inclusion criteria for this study were as follows: *Nocardia* isolation from qualified sputum specimens with at least two positive cultures, or from lung tissue or bronchoalveolar lavage fluid (BALF) obtained under sterile operating conditions, along with the presence of clinical signs of infection and/or radiographic evidence (pulmonary, skin, and soft tissue) of organ involvement. The exclusion criteria included patients with only one positive sputum culture and cases where all *Nocardia* isolates originated from blood agar plate (BAP) culture and BACTEC-MGIT 960 culture system (MGIT960).

### Identification of strains

Suspected *Nocardia* specimens found in smear microscopy of clinical samples were inoculated onto BAP and incubated in a (35 ± 2) °C incubator for 1–7 days, with daily observations of growth. *Nocardia* identification was carried out based on colony morphology on the culture medium, positive Gram stain (Gram-positive branching, moniliform, and filamentous bacilli), acid-fast staining, and modified acid-fast staining for preliminary presumptive identification. Further species identification was performed using MALDI-TOF MS. In cases where MALDI-TOF MS failed to provide species-level identification, final identification was achieved through polymerase chain reaction (PCR) amplification and sequencing of the full length of the 16S rRNA gene. The 16S rRNA primers used were: forward primer (5'-AGAGTTTGATCCTGGCTCAG-3'), reverse primer (5'-CGGTTACCTTGTTACGACTT-3'), with an amplification length of approximately 1500 bp. The amplified products were purified by Sangon Biotech (Shanghai, China) and sequenced using an ABI 3730XL gene sequencer. At the species level, the identity of the PCR products was confirmed by searching the 16S rRNA gene sequence in the NCBI GenBank using BLAST software (http://www.ncbi.nlm.nih.gov). *Nocardia* isolates were considered identified at the species level when similarity values ≥ 99.0% were obtained.

### Antimicrobial susceptibility testing

Antibiotic susceptibility testing (AST) was performed using the microbroth dilution method provided by Thermo Fisher (USA). In brief, colonies growing on BAP were collected using a swab and suspended in deionized water. The turbidity was adjusted to a 0.5 McFarland standard by visual inspection or with the help of a Sensititre® Turbidimeter. Next, 50μL of the bacterial suspension was transferred into a test tube containing cation-adjusted Mueller–Hinton broth medium with TES buffer added, and mixed thoroughly. Subsequently, 100 μL of the bacterial suspension was transferred onto a drug susceptibility plate and incubated at 35℃ for 72 h. The results were then observed and the minimum inhibitory concentration (MIC) was determined based on the instructions provided on the drug susceptibility plate. Interpretations of the results were made according to clinical and laboratory standards institute (CLSI) recommendations for *Nocardia*. Quality control strains, *Staphylococcus aureus* ATCC29213 and *Escherichia coli* ATCC35218, were included in the testing. The MIC was defined as the lowest concentration of a drug that inhibited visible growth.

### Comparison between drug sensitivity data

Accurate identification of* Nocardia* species provides the potential to partially predict antimicrobial susceptibility and helps in the selection of appropriate therapeutic approaches, for this reason we we reviewed the literature with drug susceptibility data between 2014–2023, from which we chose four representative papers (large size, large number, reliable drug susceptibility methods, etc.) to do a cross-sectional comparative study with our findings and with CLSI M62 in the The results were compared with our study in a cross-sectional study and with the CLSI M62 drug susceptibility patterns of different *Nocardia* species to find out the similarities and differences between them.

### Statistical analysis

The MIC data for each antibiotic were recorded and analyzed using WHONET 5.6 software. The MIC50 and MIC90 were calculated as well. Additionally, data analysis was performed using SPSS 25.0 statistical software. Normally distributed measurement data were presented as mean ± standard deviation (x ± s), while non-normally distributed measurement data were presented as the median. A comparison was made between patients with bronchiectasis and those non-bronchiectasis in terms of the distribution, drug susceptibility, and clinical characteristics of *Nocardia* strains. Categorical variables were compared using the x^2^ test or Fisher exact test.

## Results

### Demographic characteristics and geographical distribution

**Fig. 1:**
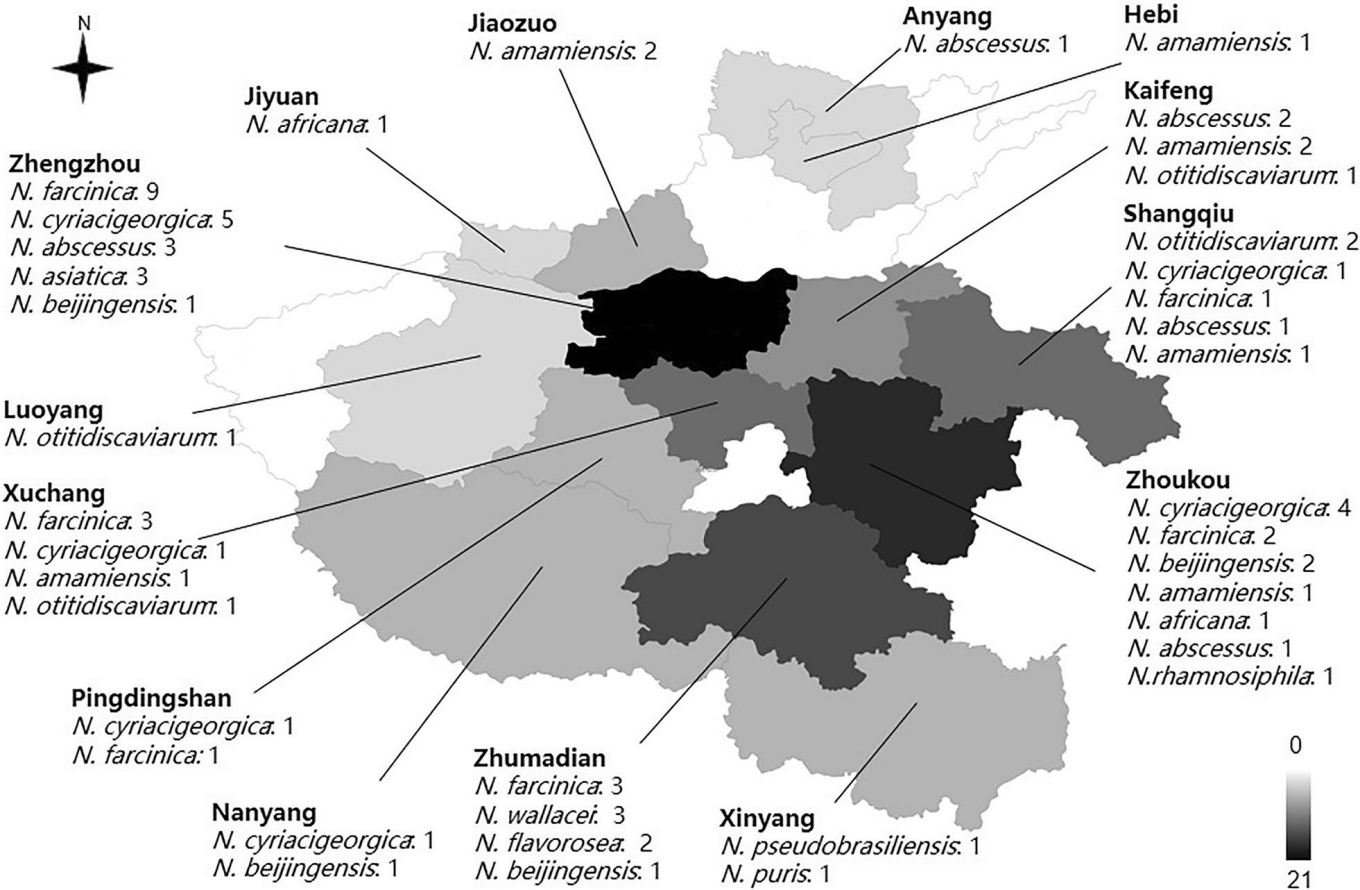
Geographical and spatial distribution of *Nocardia* isolates in Henan Province,China

A total of 71 cases of nocardiosis were collected, with ages ranging from 18 to 85 years and an average age of 56 years. Among these cases, 31 (43.6%) were aged 60 years or older, including 42 males and 29 females. The majority of the patients were farmers (67.6%, 48), and the main department involved was respiratory medicine (60.6%, 43). The most common specimen sources were sputum (52.1%, 37) and alveolar lavage fluid (46.5%, 33). Please refer to Table 1 for more details. The geographical distribution of *Nocardia* is depicted in Fig. 1. The city with the highest number of sources was Zhengzhou (21), followed by Zhoukou (12), Zhumadian (9), Shangqiu and Xuchang (6 strains each), and the classification of *Nocardia* species in various municipalities shows different.

**Table 1:** Basline characteristics of included patients

Characteristics	N	(%)
Mean age (range) (ys)	56(18–85)	
< 40	13/71	18.3
40–60	27/71	38
≥ 60	31/71	43.6
Male sex	42/71	59.2
Occupational distribution		
Farmer	48/71	67.6
Male sex	29/48	60.4
Urban workers	18/71	25.4
Others	5/71	7
Departmental distribution of isolated strains		
Respiratory department	43/71	60.6
Tuberculosis departmen	17/71	23.9
Thoracic surgery	6/71	8.5
Outpatient	4/71	5.6
Cardiology department	1/71	1.4
Infection types and sample sources		
Pulmonary nocardiosis		
Sputum	37/71	52.1
Bronchoalveolar lavage fluid	33/71	46.5
*N. farcinica*	18/71	25.4
*N. cyriacigeorgica*	13/71	18.3
*N. abscessus*	8/71	11.3
*N. amamiensis*	8/71	11.3
*N. beijingensis*	5/71	7
*N. otitidiscaviarum*	5/71	7
*N. wallacei*	3/71	4.2
*N. asiatica*	3/71	4.2
*N. flavorosea*	2/71	2.8
*N. africana*	2/71	2.8
*N. rhamnosiphila*	1/71	1.4
*N.pseudobrasiliensis*	1/71	1.4
*N. puris*	1/71	1.4
Skin and subcutaneous nocardiosis		
Skin and soft tissue pus	1/71	1.4
*N. farcinica*	1/71	1.4
Smoking history	17/71	23.9
History of drinking	9/71	12.7
Diseases history		
Yes/No	64/7	90.1/8.9
Underlying diseases		
Bronchiectasis	39/71	54.9
Pulmonary tuberculosis	16/71	22.5
Type 2 diabetes mellitus	12/71	16.9
COPD	8/71	11.3
Hypertension	8/71	11.3
Anemia	5/71	7
Coronary heart disease	3/71	4.2
Sjogren's syndrome	1/71	1.4
Chest radiograph		
Nodular or consolidative opacities	66/66	100
Cavitary lesion	16/66	24.2
Pleural effusion	21/66	31.8
Laboratory data		
WBC increased	22/65	33.8
Increased proportion of NEU(%)	39/65	60
C-reactive protein elevation	29/42	69
Increased ESR	24/31	77.4
Co-infection		
Yes/No	37/34	52.1/47.9
MTB	16/37	43.2
Fungus (aspergillosis *spp*)	5/37	13.5
NTM	2/37	.5.4
Other bacteria	18/37	48.6
Treatment Plan		
TMP-SMX + One antibiotic	10/61	16.4
TMP-SMX + Two or more antibiotics	22/61	36.1
Amikacin + Other antibiotics	20/61	32.8
Linezolid + Other antibiotics	20/61	32.8
Quinolones + Other antibiotics	24/61	39.3
Other antibiotic treatments	17/61	27.9
Outcome		
Unknow	3/71	4.2
Failer	10/68	14.7
Recovered	58/68	85.3

*COPD* chronic obstructive pulmonary Disease

*WBC* white blood cell NEU,neutrophils

*ESR* erythrocyte sedimentation rate

*MTB* Mycobacterium tuberculosis

*NTM* nontuberculosis mycobacteria

*TMP-SMX* trimethoprim-sulfamethoxazol

### Clinical characteristics

Among patients infected with *Nocardia*, 23.9% had a history of smoking, and 12.7% had a history of alcohol consumption. Out of 71 patients, 90.1% had at least one underlying disease. These included bronchiectasis (39), tuberculosis(16), type 2 diabetes (12), chronic obstructive pulmonary disease (COPD)(8), hypertension (8),anemia (5), coronary heart disease (3) and Sjogren's syndrome (1). During the diagnostic process for nocardiosis, 66 patients underwent complete imaging, where all CT scans revealed the presence of nodules or consolidative opacities. Lung cavity lesions were present in 24.2% of patients (16/66) and pleural effusions were observed in 31.8% of patients (21/66).Blood cell examinations were conducted on 65 patients. Among them, 33.8% (22/65) had elevated white blood cell counts, and 60% (39/65) had increased neutrophil proportions.CRP examinations were performed on 42 patients, and among them, 69% (29/42) showed elevated CRP levels. Additionally, among the 31 patients who underwent ESR examination, 24 had elevated ESR levels. Concurrent infections with other pathogens were present in 37 patients. These included 16 cases of *Mycobacterium tuberculosis*(MTB) infection, 5 cases of *Aspergillus.spp* infection, and 2 cases of non-tuberculous mycobacterium infection.Please refer to Table 1 for further details.

### Molecular identification and distribution of *Nocardia* species

We initially identified the *Nocardia* species using MALDI-TOF MS and confirmed it further through 16S rRNA sequencing. However, the results of MALDI-TOF MS identification for the rare *Nocardia* species (*N. africana, N.pseudobrasiliensis, N.flavorosea, N.amamiensis* and *N.rhamnosiphila*) were inadequate and inconsistent with the sequencing results. Conversely, the identification results for *N.cyriacigeorgica*,*N. farcinica*,*N. abscessus, N.beijingensis*,*N. otitidiscaviarum*,*N. asiatica*,*N. puris* and *N.wallacei* were generally consistent between the two methods.

**Fig. 2:**
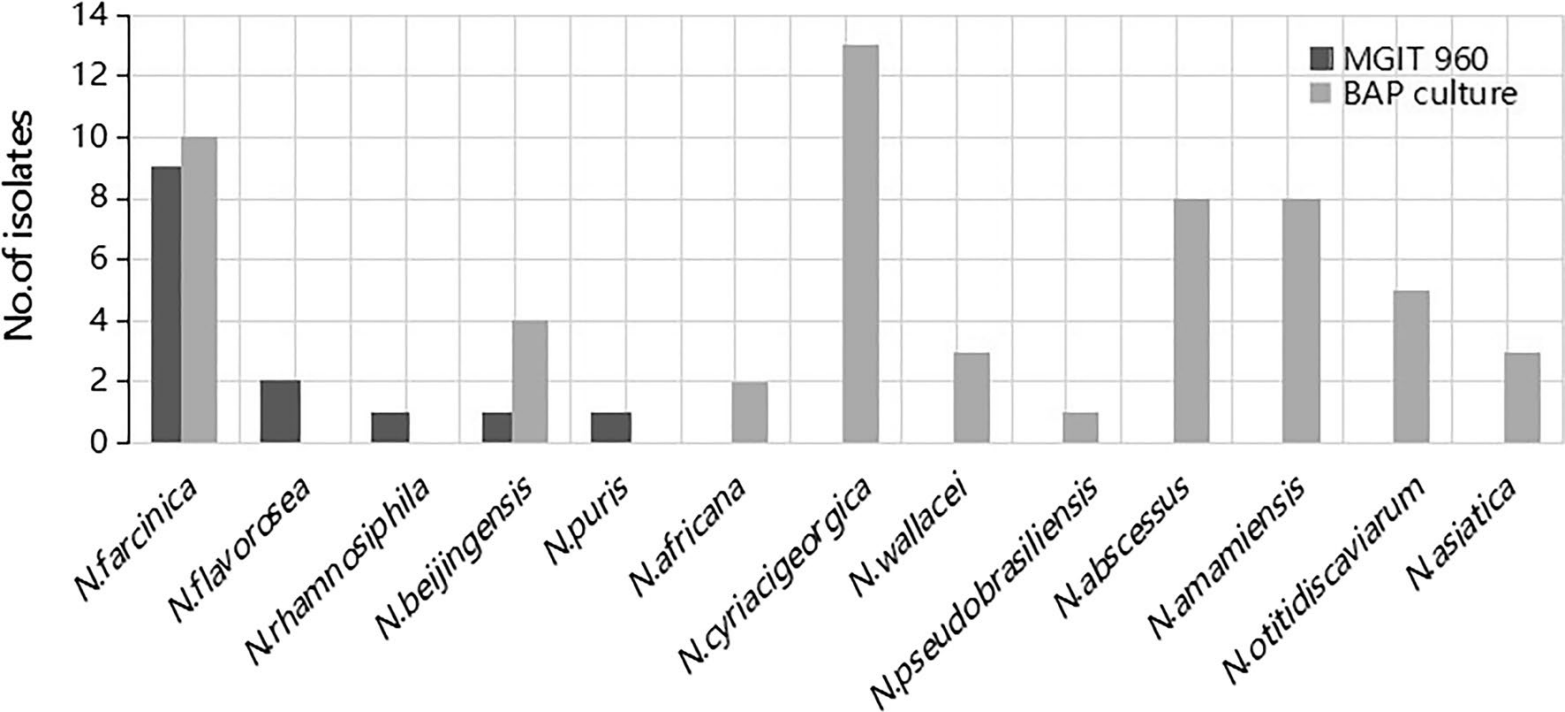
Distribution of *Nocardia* strains isolated by blood agar plate (BAP) culture and BACTEC MGIT 960 culture system (MGIT 960)

In the Additional file 1, we provide details of annual *Nocardia* isolates.The detection rate of *Nocardia* has exhibited an upward trajectory, increasing from a single isolate in 2017 to 20 cases in 2022. Among the 71 collected *Nocardia* isolates, a total of 13 species were identified. The predominant *Nocardia* species comprised *N. farcinica*(26.8%,9),*N.cyriacigeorgica*(18.3%,13),*N.abscessus*(11.3%,8), *N.amamiensis* (11.3%,8), *N.otitidiscaviarum* (7.0%,5)and *N.beijingensis* (7.0%,5). In our study, *Nocardia* strains were obtained using both traditional culture methods (BAP) and the MGIT960 culture method. Notably, there were disparities in *Nocardia* species detection between these two methods, as visually represented in Fig. 2. *N. farcinica* was the most frequently detected species via MGIT960 culture, while *N. cyriacigeorgica* predominated in traditional BAP culture.

### Antibiotic sensitivity

Table 2 provides a summary of *Nocardia*'s sensitivity to 15 antibiotics, including the MIC inhibiting 50% (MIC50) and 90% (MIC90) of strains, along with the MIC range for all *Nocardia* isolates.It displays the sensitivity rate, intermediate rate, and resistance rate for each antibiotic. All *Nocardia* strains were 100% sensitive to TMP-SMX and linezolid. It is worth noting that different species of *Nocardia* demonstrate different antibiotic resistances, as shown in Table 2, *N. farcinica* has higher resistance rates to imipenem (47.4%), ceftriaxone (78.9%), tigecycline (78.9%) and clarithromycin (89.4%), while *N.cyriacigeorgica* has lower rates of resistance to imipenem(15.4%), ceftriaxone (15.4%), tigecycline (0%),and clarithromycin (23.1%) In addition, *N. farcinica* and *N. abscessus* exhibited different sensitivities to doxycycline and minocycline. One isolate of *N. pseudobrasiliensis* exhibited resistance to ceftriaxone, ciprofloxacin, doxycycline and minocycline, and intermediate susceptibility to imipenem, moxifloxacin, and amoxicillin-clavulanic acid.

**Table 2:** Antimicrobial susceptibility and MICs of 71 *Nocardia* isolates in clinical infections in Henan China from 2017 to 2023

Drugs Breakpoint		Species/complex, no. of strains (%)^a^										
		*N.farcinica,*	*N.abscessus* complex,16 (22.5)^b^	*N. cyriacigeorgica,*	*N.amamiensis,*	*N.otitidiscaviarum,* 5(7.0)	*N. wallacei,*	*N. africana,*	*N.flavorosea,*	*N.puris*,	*N.pseudobrasiliensis,* 1 (1.4)	*N.rhamnosiphila, *(1.4)
		19(26.8)		13,(18.3)	8(11.3)		3(4.2)	2(2.8)	2 (2.8)	1 (1.4)		
TMP-SMX (S ≤ 2/38, R ≥ 4/76)	MIC_50_	≤ 0.25/4.75	≤ 0.25/4.75	≤ 0.25/4.75	≤ 0.25/4.75	0.5/9.5	≤ 0.25/4.75					
	MIC_90_	1/19	1/19	1/19	0.5/9.5	2/38	1/19					
	S/R (%)	100/0	100/0	100/0	100/0	100/0	100/0	100/0	100/0	100/0	100/0	100/0
Linezolid (S ≤ 8, ~)	MIC_50_	≤ 1	≤ 1	≤ 1	≤ 1	≤ 1	≤ 1					
	MIC_90_	2	≤ 1	≤ 1	≤ 1	2	≤ 1					
	S/NS (%)	100/0	100/0	100/0	100/0	100/0	100/0	100/0	100/0	100/0	100/0	100/0
Ciprofloxacin (S ≤ 1, R ≥ 4)	MIC_50_	1	> 4	> 4	> 4	2	1					
	MIC_90_	4	> 4	> 4	> 4	4	2					
	S/I/R (%)	78.9/10.5/10.5	18.8/18.8/62.5	0/0/100	0/0/100	20/40/40	66.7/33.3/0	0/50/50	100/0/0	0/0/100	0/0/100	100/0/0
Imipenem (S ≤ 4, R ≥ 16)	MIC_50_	8	4	≤ 2	4	64	8					
	MIC_90_	32	64	16	8	> 64	> 64					
	S/I/R (%)	26.3/26.3/47.4	50/0/50	61.5/23.1/15.4	75/25/0	0/0/100	33.3/33.3/33.3	0/0/100	100/0/0	100/0/0	0/100/0	100/0/0
Moxifloxacin (S ≤ 1, R ≥ 4)	MIC_50_	0.5	8	2	4	1	≤ 0.25					
	MIC_90_	2	16	4	8	2	1					
	S/I/R (%)	84.2/10.5/5.3	25/18.8/56.2	7.7/61.5/30.8	0/12.5/87.5	60/40/0	100/0/0	100/0/0	100/0/0	100/0/0	0/100/0	100/0/0
Cefepime	MIC_50_	> 32	4	16	4	> 32	4					
	MIC_90_	> 32	16	> 32	> 32	> 32	32					
	Range	16 to > 32	≤ 1 to 32	2 to > 32	2 to > 128	~ > 32	4 to 32					
AUG (S ≤ 8/4, R ≥ 32/16)	MIC_50_	8/4	16/8	16/8	16/8	> 64/32	8/4					
	MIC_90_	16/8	> 64/32	32/16	16/8	> 64/32	16/8					
	S/I/R (%)	52.6/42.1/5.3	43.8/6.2/50	0/53.8/46.2	37.5/62.5/0	0/0/100	66.7/33.3/0	0/0/100	50/0/50	0/0/100	0/100/0	0/0/100
Amikacin (S ≤ 8, R ≥ 16)	MIC_50_	≤ 1	≤ 1	≤ 1	≤ 1	≤ 1	2					
	MIC_90_	≤ 1	≤ 1	≤ 1	2	≤ 1	16					
	S/I/R (%)	100/0/0	100/0/0	100/0/0	100/0/0	100/0/0	66.7/0/33.3	100/0/0	100/0/0	100/0/0	100/0/0	100/0/0
Ceftriaxone (S ≤ 8, R ≥ 64)	MIC_50_	64	4	≤ 4	≤ 4	> 64	8					
	MIC_90_	> 64	8	64	64	> 64	32					
	S/I/R (%)	5.3/15.8/78.9	93.8/6.2/0	76.9/7.7/15.4	87.5/0/12.5	0/20/80	66.7/33.3/0	0/0/100	50/50/0	100/0/0	0/0/100	100/0/0
Doxycycline (S ≤ 1, R ≥ 8)	MIC_50_	2	0.12	2	≤ 0.12	1	2					
	MIC_90_	4	0.5	2	2	2	2					
	S/I/R (%)	26.3/73.7/0	93.8/6.2/0	46.2/53.8/0	87.5/12.5/0	60/40/0	0/100/0	0/100/0	50/50/0	100/0/0	0/0/100	100/0/0
Minocycline (S ≤ 1, R ≥ 8)	MIC_50_	2	≤ 1	≤ 1	≤ 1	≤ 1	2					
	MIC_90_	2	≤ 1	2	≤ 1	2	2					
	S/I/R (%)	36.8/63.2/0	93.8/6.2/0	61.5/38.5/0	100/0/0	80/20/0	33.3/66.7/0	50/50/0	50/50/0	100/0/0	0/0/100	100/0/0
Tobramycin (S ≤ 4, R ≥ 16)	MIC_50_	16	≤ 1	≤ 1	≤ 1	2	> 16					
	MIC_90_	> 16	≤ 1	≤ 1	8	4	> 16					
	S/I/R (%)	5.3/15.8/78.9	100/0/0	100/0/0	87.5/12.5/0	100/0/0	33.3/0/66.7	100/0/0	100/0/0	100/0/0	100/0/0	100/0/0
Clarithromycin (S ≤ 2, R ≥ 8)	MIC_50_	> 16	1	2	0.06	16	0.5					
	MIC_90_	> 16	> 16	> 16	4	> 16	16					
	S/I/R (%)	5.3/5.3/89.4	56.2/6.2/37.5	69.2/7.7/23.1	87.5/12.5/0	20/20/60	66.7/0/33.3	0/0/100	50/50/0	0/0/100	100/0/0	100/0/0
Cefoxitin	MIC_50_	64	8	64	16	> 128	64					
	MIC_90_	> 128	16	> 128	> 128	> 128	128					
	Range	32 to > 128	≤ 4 to 128	16 to > 128	≤ 4 to > 128	128 to > 128	64 to 128					
Tigecycline	MIC_50_	0.5	0.25	0.25	0.06	0.25	0.25					
	MIC_90_	2	0.5	1	0.25	0.5	0.5					
	Range	0.03 to 4	≤ 0.015 to 1	0.12 to 1	0.03 to 0.25	0.06 to 0.5	0.25 to 0.5					

*Nocardia* species/complex responsible for clinical infections in Henan China from 2017 to 2023. TMP-SMX, trimethoprim-sulfamethoxazole. AUG, Amoxicillin-clavulanic acid

S, susceptible; I, intermediate; R, resistant; NS, nonsusceptible; MIC50 and MIC90,MICs at which 50% and 90% of the strains were inhibited, respectively

The table shows the antimicrobial susceptibilities profifiles and MIC values (in *m*g/mL) (as determined by the broth microdilution method) to 15 antibiotics of the major

^a^Percentage with respect to the total number of identifified *Nocardia* strains (n = 71)

^b^*N. abscessus complex* (16) includes *N. abscessus* (8), *N. asiatica* (3), and *N. beijingensis* (5)

The drug sensitivity results for each *Nocardia* species in this study were compared with the predicted antimicrobial drug sensitivity patterns provided by the CLSI standard M62 and larger-scale data studies. Table 3 showed a strong correlation between the drug pattern types and the identification of *Nocardia* species. Nonetheless, some differences were observed.For example, although only 52.6% of the cutaneous *Nocardia* isolates were sensitive to amoxicillin-clavulanate, the drug pattern indicated sensitivity according to the CLSI M62.The sensitivity rate of amoxicillin-clavulanate in *N.abscessus* complex isolates was only 43.8%, despite the drug pattern indicating sensitivity.The sensitivity rate of ciprofloxacin in *N.otitidiscaviarum* isolates was 20%, with the drug sensitivity pattern also indicating sensitivity.Furthermore, despite the sensitivity rate of 100% for amikacin in *N.wallacei* isolates, the drug sensitivity pattern indicated resistance.

**Table 3:** Main results of antibiotic susceptibility testing of clinical *Nocardia* isolates, derived from large-scale studies published beteen 2014 and 2023

% of susceptible isolates													
First author, year	AST method	Break-points	N of isolates	Amikacin	AUG	Ceftriaxone	Ciprofloxacin	Imipenem	Linezolid	Minocycline	Moxifloxacin	TMP-	Tobramycin
												SMX	
All *Nocardia* isolates													
This study	BMD	CLSI	71	98.6	32.4	53.5	33.8	45.1	100	66.2	46.5	100	70.4
Hao Wang, 2022 [7]	BMD	CLSI	441	99.3	39.5	40.6	33.6	43.3	100	43.5	57.1	99.1	56.5
Hamdi, 2020 [8]	BMD	CLSI	2091	94	44	36	16	73	100	30	30	98	52
Schlaberg, 2014 [9]	BMD	CLSI	1299^b^	95	37	56	17	49	100	22	40(n = 642)	98	55
Jing Yang, 2023 [10]	BMD	CLSI	130	100	35.4	48.5	17.7	43.9	100	63.9	48.5	97.7	74.6
*N.farcinica* (N.farcinica^a^)				S	S	R	S	V	S	V	N/A	S	R
This study	BMD	CLSI	19	100	52.6	5.3	78.9	26.3	100	36.8	84.2	100	5.3
Hao Wang, 2022 [7]	BMD	CLSI	176	100	61.9	8.5	68.8	39.8	100	26.1	90.3	97.7	14.2
Hamdi, 2020 [8]	BMD	CLSI	319	100	96	3	49	83	100	7	76	99	1
Schlaberg, 2014 [9]	BMD	CLSI	204^b^	100	76	3	43	33	100	5	79(n = 99)	99	0
Jing Yang, 2023 [10]	BMD	CLSI	27	100	66.7	7.4	59.3	29.7	100	29.6	92.6	92.6	14.8
*N.abscessus* (*N.abscessus* complex^a^)				S	S	S	R	V	S	V	N/A	S	V
This study	BMD	CLSI	16	100	43.8	93.8	18.8	50	100	93.8	25	100	100
Hao Wang,2022 [7]	BMD	CLSI	54	100	48.1	90.7	18.5	46.3	100	79.6	29.6	100	87
Hamdi, 2020 [8]	BMD	CLSI	205	100	61	93	3	64	100	93	13	100	100
Schlaberg, 2014 [9]	BMD	CLSI	110^b^	100	78	98	0	31	100	85	8(n = 39)	100	100
Jing Yang, 2023 [10]	BMD	CLSI	20	100	55	95	10	55	100	85	10	100	90
*N.cyriacigeorgica* (*N.cyriacigeorgica*^a^)				S	R	S	R	S	S	V	N/A	S	S
This study	BMD	CLSI	13	100	0	76.9	0	61.5	100	61.5	7.7	100	100
Hao Wang, 2022 [7]	BMD	CLSI	126	100	6.3	66.7	2.4	58.7	100	42.9	18.3	100	96
Hamdi, 2020 [8]	BMD	CLSI	352	99	8	64	0	99	100	14	1	100	99
Schlaberg, 2014 [9]	BMD	CLSI	264^b^	100	3	88	0	43	100	6	4(n = 128)	100	99
Jing Yang, 2023 [10]	BMD	CLSI	49	100	6.1	67.3	0	61.2	100	63.3	22.4	100	100
*N.otitidiscaviarum* (*N.otitidiscaviarum*^a^)				S	R	R	S	R	S	V	N/A	S	V
This study	BMD	CLSI	5	100	0	0	20	0	100	80	60	100	100
Hao Wang, 2022 [7]	BMD	CLSI	26	100	11.5	3.8	7.7	3.8	100	61.5	46.2	100	57.7
Hamdi, 2020 [8]	BMD	CLSI	30	100	0	0	0	3	100	60	23	87	53
Schlaberg, 2014 [9]	BMD	CLSI	29^b^	100	0	0	7	7	100	45	35 (n = 17)	100	62
Jing Yang, 2023 [10]	BMD	CLSI	14	100	7.1	7.1	7.2	0	100	61.5	57.1	100	78.6
*N.wallacei (N.transvalensis* complex^a^)				R	V	S	S	V	S	V	N/A	S	R
This study	BMD	CLSI	3	100	66.7	66.7	66.7	33.3	100	33.3	100	100	33.3
Hao Wang, 2022 [7]	BMD	CLSI	11	72.7	63.6	63.6	72.7	36.4	100	27.3	90.9	100	9.1
Hamdi, 2020 [8]	BMD	CLSI	121	26	89	64	49	9	100	31	72	88	0
Schlaberg, 2014 [9]	BMD	CLSI	83	28	47	63	84	6	100	15	100	81	4
Jing Yang, 2023 [10]	BMD	CLSI	3	100	N/A	N/A	N/A	N/A	100	N/A	N/A	N/A	N/A

*AST* antimicrobial susceptibility testing, *BMD* broth microdilution, *CLSI* Clinical and Laboratory Standards Institute, *N* number, *N/A* not available

^a^Expected antimicrobial susceptibility patterns of the most com monly isolated *Nocardia* species or species complexes provided by CLSI standard M62 [11]; the expected pattern “R/S/V” represents resistant/susceptible/variable

^b^Except for moxifloxacin

### Comparison between pulmonary nocardiosis complicated with bronchiectasis group and non-bronchiectasis group

Bronchiectasis was found to be the predominant underlying disease among the patients based on the data presented in Table 1. The collected cases of nocardiosis were classified into two groups: the bronchiectasis group (39) and the non-bronchiectasis group(29), based on the patients' radiological examination results. We conducted a comprehensive comparison of the clinical characteristics, distribution of bacterial species, and drug sensitivity results between these two groups.Further detailed comparisons are provided in Tables 4 and 5.

**Table 4:** Characteristics of patients with nocardiosis

Characteristics	Bronchiectasis (n = 39)^a^	Non bronchiectasis	P value*
		(n = 29)^a^	
Patient demographics			
Male/female	18/21	8/21/2023	**0.030**
< 40 years	8 (20.5%)	5 (17.2%)	0.734
40–60 years	13 (33.3%)	14 (48.3%)	0.213
≥ 60 years	18 (46.2%)	10 (34.5%)	0.333
Smoking history	6 (15.4%)	11 (37.9%)	**0.034**
History of Drinking	3 (7.7%)	6 (20.7%)	0.156
Underlying diseases			
Healthy	6 (15.4%)	1 (3.4%)	0.225
COPD	5 (12.8%)	3 (10.3%)	1.000
Diabetes	3 (7.7%)	9 (31.0%)	**0.013**
Hypertension	2 (5.1%)	6 (20.7%)	0.065
Pulmonary tuberculosis	6 (15.4%)	10 (34.5%)	0.066
Coronary heart disease	0 (0%)	3 (10.3%)	0.073
Anemia	2 (5.1%)	3 (10.3%)	0.644
Chest radiograph			
Nodular or consolidative opacities	38 (97.4%)	28 (96.5%)	1.000
Cavitary lesion	6 (15.4%)	10 (34.5%)	0.066
Pleural effusion	8 (20.5%)	13 (44.8%)	**0.032**
Co-infection			
MTB	4 (10.3%)	10 (34.5%)	**0.015**
NTM	1 (2.6%)	1 (3.4%)	1.000
Fungus (*aspergillosis spp.*)	1 (2.6%)	4 (13.8%)	0.155
Other bacteria	11 (28.2%)	7 (24.1%)	0.707
Outcome			
Failer	1 (2.6%)	9 (31.0%)	**0.001**
Cure	38 (97.4%)	20 (69.0%)	**0.001**

*Due to total number < 40 and some of the expected number < 5, Fisher exact test’s result was used

^a^Three patients were lost to follow-up (including two pulmonary and one cutaneous), whose presence or absence of bronchiectasis was unknown due to incomplete case data

**Table 5:** *Nocardia* species identification and antibiotic risistance

Characteristics	Bronchiectasis	Non bronchiectasis	P value*
	(n = 39)^a^	(n = 29)^a^	
*Nocardia* species identifification			
*N.cyriacigeorgica*	9 (23.1%)	3 (10.3%)	0.173
*N.abscessus*	8 (20.5%)	0 (0%)	**0.017**
*N.farcinica*	6 (15.4%)	12 (41.4%)	**0.016**
*N.amamiensis*	6 (15.4%)	2 (6.9%)	0.451
*N.otitidiscaviarum*	4 (10.3%)	1 (3.4%)	0.384
*N.beijingensis*	2 (5.1%)	3 (10.3%)	0.644
*N.asiatica*	1 (2.6%)	2 (6.9%)	0.571
*N.puris*	1 (2.6%)	0 (0%)	1.000
*N. wallacei*	1 (2.6%)	2 (6.9%)	0.571
*N.pseudobrasiliensis*	1 (2.6%)	0 (0%)	1.000
*N.flavorosea*	0 (0%)	1 (3.4%)	0.426
*N.rhamnosiphila*	0 (0%)	1 (3.4%)	0.426
*N.africana*	0 (0%)	2 (6.9%)	0.178
Antibiotic resistance profifiles			
AUG	14 (35.9%)	10 (34.5%)	0.904
Ceftriaxone	9 (23.1%)	15 (51.7%)	**0.014**
Imipenem	14 (35.9%)	12(41.4%)	0.645
Tobramycin	3 (7.7%)	13 (44.8%)	**0.000**
Ciprofloxacin	25 (64.1%)	12 (41.4%)	0.063
Moxifloxacin	11 (28.2%)	10 (34.5%)	0.799
Amikacin	0 (0%)	1 (3.4%)	0.426
Linezolid	0 (0%)	0 (0%)	> 1.000
TMP-SMX	0 (0%)	0 (0%)	> 1.000
Clarithromycin	16 (41%)	15 (51.7%)	0.381
Doxycycline	1(2.6%)	0 (0%)	1.000
Minocycline	1 (2.6%)	0 (0%)	1.000

*Due to total number < 40 and some of the expected number < 5, Fisher exact test’s result was used

^a^Three patients were lost to follow-up (including two pulmonary and one cutaneous), whose presence or absence of bronchiectasis was unknown due to incomplete case data

It can be concluded from Table 4 that there was no statistically significant difference in the distribution of age groups between the two groups (p > 0.05), but in terms of gender composition, the bronchiectasis group was predominantly female, whereas the non-bronchiectasis group was predominantly male. There was a statistical difference between the two groups in terms of smoking history, with patients with a history of smoking in the non-bronchodilated group being more susceptible to nocardiosis than those with a history of smoking in the bronchodilated group. In terms of comorbidities between the two groups, we learnt that patients with diabetes mellitus in the non-bronchiectasis group were more likely to develop nocardiosis. Other differences are detailed in Table 4.

In Table 5, our study found that there was a statistical difference between the two groups in terms of species distribution of *Nocardia* isolates, in both *N.abscessus* (p < 0.05) and *N.farcinica* (p < 0.05) detection rates, with patients with bronchodilatation being more likely to be detected with *N. abscessus* than those with non-bronchodilatation. In antibiotic susceptibility testing, there was a statistical difference between the two groups in ceftriaxone and tobramycin (p < 0.05) antimicrobial drugs.

### Treatment and outcome

Out of the 71 cases of nocardiosis that were collected, treatment details were available for 61 patients, while the treatment plans for 10 patients were missing.Based on the data presented in Table 1, out of the 61 patients with treatment plans, 32 patients (52.5%) received combination therapy comprising of TMP-SMX. Among these patients, 10 received TMP-SMX combined with a single drug, while 22 patients received TMP-SMX combined with two or more antibiotics as part of a multidrug treatment plan.Moreover, 20 patients (32.8%) received a multidrug regimen containing amikacin and other antibiotics, while 20 patients (32.8%) received a regimen consisting of linezolid combined with other antibiotics. Furthermore, 24 patients (39.3%) were treated with a combination of quinolone antibiotics and other antibiotics, and 17 patients were prescribed alternative antibiotic regimens during the treatment. Out of the 71 patients' treatment outcomes, 3 patients were lost to follow-up with unknown treatment results, 58 patients (85.3%) achieved a cure or improvement in clinical symptoms, and 10 patients (14.7%) were discharged without being cured.

## Discussion

*Nocardia*, an important group of actinomycetes in the environment, can lead to human infections through traumatic inoculation or inhalation [1]. The latest taxonomic study of *Nocardia* reveals the existence of 119 species, with 54 of them being associated with human infections [12, 13]. Some medically relevant bacterial species include *N. asterosa*, *N.brasiliensis*, *N.farcinica a*nd *N.abscessus*. Over the past decade, China has witnessed an increasing trend in *Nocardia* infections [7, 10, 14, 15]. However, there is a scarcity of reports on *Nocardia* in Henan. Given the variation in *Nocardia* distribution across different geographical regions, it is essential to investigate the epidemiology, clinical characteristics, and antibiotic resistance of *Nocardia* in various areas.

The incidence of nocardiosis is influenced by age. Our data showed that the majority of patients were older with a slight male predominance, which is consistent with previous literature [7, 10]. Occupational classification was dominated by farmers, who may be more susceptible to environmental *Nocardia* infections due to their greater outdoor exposure to contaminated soil.In our study, the diagnostic methods for *Nocardia* infection include BAP culture and MGIT960 culture. As shown in Fig. 2, *N.farcinica* was most frequently detected through the MGIT 960 culture method (n = 9, 60%), which is consistent with the literature [16, 17]. A study by Hu et al. [16] found that the high recovery rate of *N.farcinica* in MGIT 960 and the growth of other *Nocardia* species are due to their resistance to trimethoprim-sulfamethoxazole. The main species of *Nocardia* varies from region to region, in Australia [18] and USA [9], the most common is *N. nova* complex. in Iran [19], France [20] and Japan [21], the most prevalent species are *N.asteroides, N.farcinica* and *N.cyriacigeorgica*. Even in China, the distribution characteristics of *Nocardia* vary from region to region. In Taiwan, China [22] and Hebei province, China [10], the most frequently occurring species are *N.brasiliensis* and *N.cyriacigeorgica*.

*Nocardia* is mainly transmitted by inhalation and pulmonary nocardiosis usually affects frail patients, especially immunocompromised patients due to organ transplantation and/or treatment with corticosteroids and COPD patients, and forms infected lesions in the lungs [23, 24]. Bronchiectasis was the most common (54.9%) among the patients with pulmonary nocardiosis combined with the underlying disease in our study. In a study by Huang et al. [25]. bronchiectasis was most common (30.4%) among the underlying diseases.In a study by Yang et al. bronchiectasis was comorbid in 6 out of 12 (50%) patients diagnosed with nocardiosis [26]. A study in Taiwan reported that the most common comorbidities were diabetes mellitus (30%) and COPD (26.7%) [27], which is similar to our report. Data show that an increasing number of patients with bronchiectasis are being diagnosed with pulmonary nocardiosis, but the reasons for this are not fully understood and may be due to environmental exposures, microbiological surveillance, and other factors [5].Woodworth et al. concluded that *N.nova c*omplex was more likely to be detected in patients with bronchiectasis than in other patients [5], but in our study data (Table 5) *N.abscessus* was more likely to be detected in patients with bronchiectasis, which is clearly inconsistent.

In this study, the clinical symptoms and CT manifestations of patients with pulmonary nocardiosis lacked specificity, making it difficult to distinguish them from other diseases such as filamentous fungi (e.g., Aspergillus and trichothecenes) or mycobacterial infections [28]. Pulmonary nocardiosis may be mistaken for tuberculosis, and tuberculosis and HIV are common co-infections [29]. Of 10 patients diagnosed with nocardiosis after death, 40% were misdiagnosed with TB before death [30]. A study evaluating patients with suspected tuberculosis in Ghana found that 16.7% were co-infected with HIV and *Nocardia spp*. [31] The antibiotic regimen for nocardiosis and other diseases varies considerably, making accurate diagnosis critical to treatment.

To this day, that TMP-SMX is the main drug used for the treatment of nocardiosis, however, some studies have raised concerns about the increased resistance of *Nocardia* isolates in particular to TMP-SMX.In the study of Lebeaux et al. [20], 5.4% of isolates were insusceptible to TMP-SMX; in the study of Uhde et al., the rate of resistance to TMP-SMX was 42% [32]; these discrepancies may be caused by inter-labortory differences or differences in species distribution in different geographical regions. A multicentre study of 441 *Nocardia* strains in China showed a resistance rate to TMP-SMX of only 0.9% [7], which is more consistent with the results of this study. In Table 3 the main results of studies reported from antibiotic sensitivity testing of large-scale clinical *Nocardia* isolates published between 2014 and 2023 show a strong correlation between the type of drug pattern and the identification of *Nocardia spp*. species, with some differences between different strains and the corresponding drug susceptibility patterns; Therefore, for the optimal treatment of nocardiosis, the *Nocardia spp.* species should be identified as accurately as possible and antimicrobial drug susceptibility testing should be performed.

TMP-SMX is typically used as the drug of choice for the treatment of nocardiosis, either alone or in combination with other drugs such as amikacin, imipenem, or third-generation cephalosporins. Amikacin can be used in combination with TMP-SMX or other drugs for the treatment of critical nocardiosis [33]. Imipenem is more active than meropenem or ertapenem against most *Nocardia* [34], and the combination of amikacin and imipenem is more effective in treating cerebral and pulmonary nocardiosis than TMP-SMX alone in a mouse model [35, 36]. The combination of imipenem and cefotaxime, amikacin and TMP-SMX, imipenem and TMP-SMX, amikacin and cefotaxime, or amikacin and imipenem provided enhanced activity [37]. For most forms of nocardiosis, initial combination drug therapy is recommended [28]. Among our 61 patients with a treatment plan for pulmonary nocardiosis, TMP-SMX in combination with amikacin and linezolid was the more common regimen. Although TMP-SMX is a common treatment option for nocardiosis, some patients in our study cases opted for other effective antimicrobials due to allergy to oral sulfonamides. Linezolid has shown good clinical efficacy in *Nocardia* infections and can be recommended as an alternative therapy to TMP-SMX due to its oral availability and activity against most *Nocardia* species [38]. In our patients with nocardiosis, after aggressive clinical treatment, 14.7% of them still failed, and the failure may be due to the severity and complexity of the patient's own underlying disease.

The study has several limitations.Firstly, being a retrospective study, it carries inherent limitations of this study design,especially in terms of data loss, such as missed patient cases and loss to follow-up. Secondly, the study’s small scale may introduce biases in epidemiology and prognosis.The data may not be representative, indicating the need for further research with a larger sample size.

In summary,This is the first study on the epidemiological and clinical characteristics of nocardiosis on a larger scale in Henan, China, and describes the distribution, clinical characteristics and antibiotic drug sensitivity of the identified *Nocardia* species. Drug susceptibility varies among different *Nocardia* species, and accurate species identification and confirmation of antimicrobial susceptibility patterns are necessary in diagnosis and selection of antibiotic therapy. Pulmonary nocardiosis is prone to comorbidities with other underlying diseases such as bronchiectasis, tuberculosis, diabetes mellitus and COPD. nocardia infections are susceptible to concurrent comorbidities with a variety of pathogens such as *Mycobacterium* and *Aspergillus*. Our study also showed that bronchiectasis occurs more frequently with *Nocardia* infections, and the data from the bronchiectasis and non-bronchiectasis groups showed statistical differences in clinical characteristics and drug sensitivity. Our study adds new value to the characterisation of nocardiosis in China, and a better understanding of the characteristics of *Nocardia* infections will help physicians make better decisions in the diagnosis and treatment of nocardiosis.

### Supplementary Information

**Additional file 1.** Distribution of *Nocardia* species detected in each year (No. of isolates).

## Data Availability

The datasets used and/or analysed during the current study are available from the corresponding author upon reasonable request.
